# The acridonecarboxamide GF120918 potently reverses P-glycoprotein-mediated resistance in human sarcoma MES-Dx5 cells

**DOI:** 10.1038/sj.bjc.6690791

**Published:** 1999-11

**Authors:** H C L Traunecker, M C G Stevens, D J Kerr, D R Ferry

**Affiliations:** CRC Institute for Cancer Studies, University of Birmingham, Birmingham B15 2TA, UK; Department of Paediatric Oncology, The Birmingham Children’s Hospital NHS Trust, Birmingham B4 6NH, UK

**Keywords:** multidrug resistance, P-glycoprotein inhibitor, GF120918, MES-Dx5 cells

## Abstract

The doxorubicin-selected, P-glycoprotein (P-gp)-expressing human sarcoma cell line MES-Dx5 showed the following levels of resistance relative to the non-P-gp-expressing parental MES-SA cells in a 72 h exposure to cytotoxic drugs: etoposide twofold, doxorubicin ninefold, vinblastine tenfold, taxotere 19-fold and taxol 94-fold. GF120918 potently reversed resistance completely for all drugs. The EC_50_s of GF120918 to reverse resistance of MES-Dx5 cells were: etoposide 7 ± 2 nM, vinblastine 19 ± 3 nM, doxorubicin 21 ± 6 nM, taxotere 57 ± 14 nM and taxol 91 ± 23 nM. MES-Dx5 cells exhibited an accumulation deficit relative to the parental MES-SA cells of 35% for [^3^H]-vinblastine, 20% for [^3^H]-taxol and [^14^C]-doxorubicin. The EC_50_ of GF120918, to reverse the accumulation deficit in MES-Dx5 cells, ranged from 37 to 64 nM for all three radiolabelled cytotoxics. [^3^H]-vinblastine bound saturably to membranes from MES-Dx5 cells with a *K*_D_ of 7.8 ± 1.4 nM and a *B*_max_ of 5.2 ± 1.6 pmol mg^–1^ protein. Binding of [^3^H]-vinblastine to P-gp in MES-Dx5 membranes was inhibited by GF120918 (*K*_i_ = 5 ± 1 nM), verapamil (*K*_i_ = 660 ± 350 nM) and doxorubicin (*K*_i_ = 6940 ± 2100 nM). Taxol, an allosteric inhibitor of [^3^H]-vinblastine binding to P-gp, could only displace 40% of [^3^H]-vinblastine (*K*_i_ = 400 ± 140 nM). The novel acridonecarboxamide derivative GF120918 potently overcomes P-gp-mediated multidrug resistance in the human sarcoma cell line MES-Dx5. Detailed analysis revealed that five times higher GF120918 concentrations were needed to reverse drug resistance to taxol in the cytotoxicity assay compared to doxorubicin, vinblastine and etoposide. An explanation for this phenomenon had not been found. © 1999 Cancer Research Campaign


*British Journal of Cancer* (1999) **81**(6), 942–951
© 1999 Cancer Research Campaign
Article no. bjoc.1998.0791

### The acridonecarboxamide GF120918 potently reverses P-glycoprotein-mediated resistance in human sarcoma MES-Dx5 cells

**HCL Traunecker[1], MCG Stevens[2], DJ Kerr[1] and DR Ferry[1]**

[1]CRC Institute for Cancer Studies, University of Birmingham, Birmingham B15 2TA, UK; [2]Department of Paediatric Oncology, The Birmingham Children's Hospital NHS Trust, Birmingham B4 6NH, UK

**Summary** The doxorubicin-selected, P-glycoprotein (P-gp)-expressing human sarcoma cell line MES-Dx5 showed the following levels of resistance relative to the non-P-gp-expressing parental MES-SA cells in a 72 h exposure to cytotoxic drugs: etoposide twofold, doxorubicin ninefold, vinblastine tenfold, taxotere 19-fold and taxol 94-fold. GF120918 potently reversed resistance completely for all drugs. The $EC_{50}$s of GF120918 to reverse resistance of MES-Dx5 cells were: etoposide 7 ± 2 nM, vinblastine 19 ± 3 nM, doxorubicin 21 ± 6 nM, taxotere 57 ± 14 nM and taxol 91 ± 23 nM. MES-Dx5 cells exhibited an accumulation deficit relative to the parental MES-SA cells of 35% for [$^3$H]-vinblastine, 20% for [$^3$H]-taxol and [$^{14}$C]-doxorubicin. The $EC_{50}$ of GF120918, to reverse the accumulation deficit in MES-Dx5 cells, ranged from 37 to 64 nM for all three radiolabelled cytotoxics. [$^3$H]-vinblastine bound saturably to membranes from MES-Dx5 cells with a $K_D$ of 7.8 ± 1.4 nM and a $B_{max}$ of 5.2 ± 1.6 pmol mg$^{-1}$ protein. Binding of [$^3$H]-vinblastine to P-gp in MES-Dx5 membranes was inhibited by GF120918 ($K_i$ = 5 ± 1 nM), verapamil ($K_i$ = 660 ± 350 nM) and doxorubicin ($K_i$ = 6940 ± 2100 nM). Taxol, an allosteric inhibitor of [$^3$H]-vinblastine binding to P-gp, could only displace 40% of [$^3$H]-vinblastine ($K_i$ = 400 ± 140 nM). The novel acridonecarboxamide derivative GF120918 potently overcomes P-gp-mediated multidrug resistance in the human sarcoma cell line MES-Dx5. Detailed analysis revealed that five times higher GF120918 concentrations were needed to reverse drug resistance to taxol in the cytotoxicity assay compared to doxorubicin, vinblastine and etoposide. An explanation for this phenomenon had not been found. © 1999 Cancer Research Campaign

**Keywords:** multidrug resistance; P-glycoprotein inhibitor; GF120918; MES-Dx5 cells

P-glycoprotein (P-gp) confers cross-resistance to a variety of structurally unrelated cytotoxic drugs, e.g. epidophyllotoxins, vinca alkaloids, anthracyclines, taxanes and actinomycin D (Endicott and Ling, 1989). P-gp is a member of the ATP-binding cassette transporter family and is able to efflux cytotoxic drugs against a concentration gradient, therefore causing a cellular drug accumulation deficit, resulting in resistance. This active transport requires the hydrolysis of ATP (Gottesman and Pastan, 1993) and can be reversed in vitro by a broad range of compounds which are structurally unrelated and non-cytotoxic, e.g. verapamil, cyclosporin A, quinidine, trifluaperazine, tamoxifen and many more (Ford and Hait, 1990).

Clinical studies have shown some benefit using the weak P-gp inhibitor verapamil in addition to chemotherapy in malignant lymphomas (Miller et al, 1991) or cyclosporin A in multiple myeloma (Sonneveld et al, 1992). Serious side-effects occurring at P-gp inhibitor concentrations required to fully reverse resistance in vitro precluded widespread use of these modulators. Therefore, the development of potent P-gp inhibitors that are better tolerated, remains an important task in successfully overcoming multidrug resistance.

*Received 23 November 1998*
*Revised 4 May 1998*
*Accepted 5 May 1998*

*Correspondence to:* DR Ferry

GF120918 (also called GG918/GW0918), an acridonecarboxamide derivative, has been identified as a new potent P-gp inhibitor (Hyafil et al, 1993). At a concentration of 20 nM, activity was consistently achieved in vitro and GF120918 was found to be 100-fold more potent than verapamil (Hyafil et al, 1993). In vivo a single dose of GF120918 sensitized mice inoculated intraperitoneally with P-gp expressing xenografts (P388/Dox leukaemia or C26 colon carcinoma) to doxorubicin (Hyafil et al, 1993).

In the experiments reported in this paper we investigate the ability of GF120918 to sensitize the P-gp-expressing human Muellerian uterine sarcoma cell line MES-Dx5, which had been selected in increasing doxorubicin concentrations up to 500 nM (Harker and Sikic, 1985).

Few examples of detailed pharmacological analysis of the potency of P-gp inhibitors to modulate resistance to various cytotoxic drugs exist. One such example is the characterization of the potency of CP100 356 by Kajiji et al (1994). In the series of experiments described, detailed quantitative efforts have been made to determine inhibitor properties of GF120918 since these results could have bearing on the design of future clinical trials with P-gp inhibitors.

### MATERIALS AND METHODS

### Materials

McCoy's 5A cell culture medium was purchased from Sigma (Poole, UK) and all other cell culture reagents from Gibco-BRL

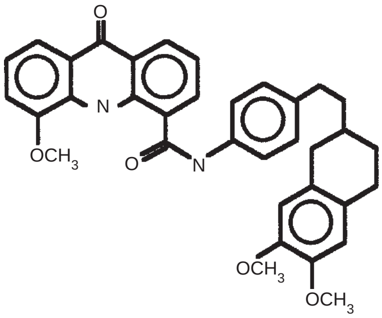

**Figure 1** Structure of GF120918

(Paisley, UK). MES-SA and MES-Dx5 were obtained from the American Type Tissue Culture Collection (Rockville, MD, USA) and MCF-7/ADR cells from the European Collection of Cell Culture (Salisbury, UK). [$^3$H]-vinblastine (11.4–13.5 Ci mmol$^{-1}$ and [$^{14}$C]-doxorubicin (60 mCi mmol$^{-1}$) were purchased from Amersham (Little Chalfont, UK). [$^3$H]-taxol (19.3 Ci mmol$^{-1}$) was obtained from the National Cancer Institute (Bethesda, MD, USA). [$^3$H]-taxol and [$^{14}$C]-doxorubicin were aliquoted and not frozen after thawing. Doxorubicin, etoposide, taxol, vinblastine and cyclosporin A were supplied by Sigma (Poole, UK). Taxotere was a gift from Rhône-Poulenc Rorer (Paris, France). Verapamil and GF120918 (Figure 1) were supplied by GlaxoWellcome (USA). CP100356 was a gift from Dr Kajiji, Pfizer (Groton, CT, USA), Dexniguldepine from Raine Boer, Byk-Gulden Lomberg GmBH (Konstanz, Germany), CGP41251 from Professor Gescher, MRC Toxicology Unit (Leicester, UK) and PSC833 from Sandoz (Basel, Switzerland). Stock solutions of drugs were made up in dimethyl sulphoxide (DMSO) at a concentration of 10 mM and stored at –20°C. Materials used for Western blotting were: monoclonal antibody NCL-JSB-1 from Novocastra Laboratories Ltd (Peterborough, UK), mouse peroxidase IgG from Sigma (Poole, UK) and enhanced chemiluminescence (ECL) protein detection reaction from Amersham (Little Chalfont, UK). Protein concentrations were measured with the Bio-Rad Protein Assay, Bio-Rad Laboratories Ltd (Hemel Hempstead, UK). All other reagents were of the highest available purity obtained from commercial sources.

### Cell culture

MES-SA cells and the doxorubicin-selected clone MES-Dx5 (Harker et al, 1983; Harker and Sikic, 1985) were grown as a monolayer in McCoy's 5A medium supplemented with 10% fetal calf serum (FCS), 20 000 U penicillin and 20 mg streptomycin per litre. The P-gp expressing MCF-7/ADR cells, derived from a human breast cancer cell line were grown as a monolayer in Dulbecco's modified Eagle's medium (DMEM) supplemented with 10% FCS, 20 000 U penicillin and 20 mg streptomycin per litre as previously described (Ferry et al, 1995). Fifty per cent FCS was employed in experiments assessing the effect of protein binding. The cells were maintained in a humidified atmosphere containing 5% carbon dioxide at 37°C. All cell lines were mycoplasma-negative.

### Cytotoxicity assay

The tetrazolium dye based MTT (3-[4,5-dimethylthiazol-2-yl]-2,5-diphenyltetrazolium bromide) assay (Mosmann, 1983) was used to assess cytotoxicity. MES-SA or MES-Dx5 cells were seeded into flat-bottom 96-well plates at 2000 cells per well and grown for 24 h in drug-free medium. Cells were incubated for 72 h with cytotoxic agents ± GF120918. A total of 20 µl of MTT dye (5 mg ml$^{-1}$ phosphate-buffered saline (PBS)) was added and incubated for 4 h at 37°C. All medium was removed and the formazan crystals dissolved in 100 µl DMSO. The absorption was measured in a spectrophotometer at a wavelength of 550 nm.

### Radiolabelled drug accumulation assay

Drug accumulation assays were performed as previously described (Ferry et al, 1995). Briefly, 80 × 10$^4$ MES-SA or MES-Dx5 cells ml$^{-1}$ were plated into each well of a 12-well plate and incubated for 24 h. McCoy's 5A medium was replaced with 1 ml uptake medium per well (McCoy's 5A medium without supplements and added 5 mM glucose and 5 mM magnesium chloride ($MgCl_2$)). The cells were incubated in a shaking waterbath at 37°C in near darkness. GF120918 (1 µM) in 10 µl of DMSO or 10 µl DMSO for the control were added to each well. [$^3$H]-vinblastine (≈ 1 nM), [$^3$H]-taxol (≈ 0.5 nM) or [$^{14}$C]-doxorubicin (≈ 20 nM) were added at a volume of 10 µl per well which commenced the assay and incubated for 30 min to 4 h at 37°C. The reaction was stopped by removal of the uptake medium and addition of 1 ml liquid scintillant. Radioactivity was quantitated by liquid scintillation counting.

### Preparation of cell surface membranes

MES-SA and MES-Dx5 cell surface membranes were prepared by a method as previously described (Ferry et al, 1992). Ten 80 cm$^2$ flasks of cells were grown to confluence (equivalent to 5 × 10$^8$ cells). After removal of medium and addition of ice-cold buffer A (50 mM Tris-HCl, pH 7.4, 0.1 mM phenylmethylsulphonyl fluoride (PMSF) and 0.1 mM EDTA) the cells were harvested with a rubber policeman. The cell suspension was centrifuged (3000 ***g***, 4°C for 3 min) and the supernatant discarded. The packed cell volume was measured and 1 ml pellet was resuspended in 9 ml ice-cold buffer A. Cells were homogenized with a polytron (6 × 30 s, setting 6). The crude homogenate was centrifuged for 10 min (3500 ***g*** at 4°C) in a Sorvall centrifuge and the supernatant was centrifuged for 30 min at 40 000 ***g*** at 4°C. The resulting membrane pellet was resuspended in 3 ml ice-cold buffer B (50 mM Tris-HCl, pH 7.4 and 0.1 mM PMSF). Aliquots of membranes were stored at –80°C and they will retain their saturable [$^3$H]-vinblastine binding activity for at least a year (Ferry et al, 1995). Protein concentrations were measured with a Bio-Rad kit using bovine serum albumin as a standard.

### Gel electrophoresis and Western blotting

A 6% sodium dodecyl sulphate (SDS) gel (Sambrook et al, 1989) was loaded with 10–100 µg membrane protein after denaturation with gel sample buffer and run at 12 mA overnight. The protein was transferred over 4 h onto a nitro-cellulose blotting membrane. Non-specific binding was blocked with 5% milk powder in PBS and Tween. Incubation with the primary monoclonal antibody NCL-JSB-1 (1:500 in 5% milk in PBS/Tween) lasted 16 h at 4°C. The secondary antibody, mouse peroxidase IgG (1:1000 in 5% milk in PBS/Tween), was added for 1 h and the protein was visualized using the ECL protein detection reaction.

*© 1999 Cancer Research Campaign*

**Table 1** $EC_{50}$ (nM) of cytotoxic drugs in MES-SA and MES-Dx5 cells (MTT dye-based cytotoxicity assay after 72-h exposure to drugs) and resistance factor for MES-Dx5 cells.

| Drug | MES-SA | | | MES-Dx5 | | | |
|---|---|---|---|---|---|---|---|
| | $EC_{50}$ (nM) | Slope | *n* | $EC_{50}$ (nM) | Slope | *n* | RF[a] |
| Etoposide | 3003 ± 1006 | 0.4 ± 0.1 | 5 | 6846 ± 504 | 1.0 ± 0.2 | 3 | 2 |
| Doxorubicin | 58 ± 14 | 0.9 ± 0.2 | 5 | 534 ± 67 | 0.9 ± 0.1 | 13 | 9 |
| Vinblastine | 3 ± 1 | 1.6 ± 0.3 | 4 | 30 ± 12 | 0.6 ± 0.1[b] | 6 | 10 |
| Taxotere | 5 ± 0.1 | 2.6 ± 0.1 | 2 | 93 ± 28 | 0.5 ± 0.1[c] | 2 | 19 |
| Taxol | 5 ± 1 | 2.0 ± 0.2 | 5 | 470 ± 98 | 0.5 ± 0.1[c] | 8 | 94 |

[a]Resistance factor; [b]$P = 0.0003$ slope MES-Dx5 vs MES-SA cells; [c]$P < 0.0001$ slope MES-Dx5 vs MES-SA cells.

### Membrane binding assay

Binding of [$^3$H]-vinblastine to membrane preparations was performed as previously described (Ferry et al, 1992). Briefly, 50 µl buffer B (50 mM Tris-HCl, pH 7.4 and 0.1 mM PMSF), 50 µl drug diluted in buffer B, 50 µl [$^3$H]-vinblastine (5–7 nM) were incubated with 100 µl membrane preparation (protein concentration 50–60 µg per assay) for 3 h in near darkness at 21°C. The total assay volume was 0.25 ml. The assay was stopped by adding 3.5 ml ice-cold washbuffer (20 mM Tris–HCl, pH 7.4 and 20 mM $MgCl_2$) followed by rapid filtration through a Whatman GF/C filter (pre-wetted with 50 mM Tris–HCl and 0.1% albumin) to separate bound from free radioactivity. The filter was washed twice with 5 ml washbuffer to remove free radioactivity, dried and added to scintillation vials filled with 3 ml scintillant. Retained, bound radioactivity was measured with a liquid scintillation counter.

### Rhodamine dye accumulation and efflux assay measured by FACS analysis

The rhodamine (Rh123) dye accumulation assay was performed as previously described (Davies et al, 1996). Briefly, $1 \times 10^6$ MES-SA or MES-Dx5 cells were seeded into 55-mm Petri dishes and allowed to become adherent overnight at 37°C. The medium was replaced with 5 ml additive free medium and incubated with 0.4 µM Rh123 for 20 min at 37°C in darkness. The cells were washed with PBS (37°C). Control cells were harvested before the dye could efflux. Additive free medium was added to the other samples and the cells were incubated for 1.5 h at 37°C. Cells were harvested with trypsin–EDTA at 4°C. Following centrifugation (3000 ***g***, 4°C for 3 min) the cell pellet was washed in ice-cold PBS and resuspended in 1 ml ice-cold PBS and propidium iodate (5 µg $ml^{-1}$), incubated for 10 min on ice followed by a wash in ice-cold PBS and centrifugation (3000 ***g***, 4°C for 3 min). The cells were fixed for 20 min in 1 ml 1% paraformaldehyde (in PBS).

Flow cytometric analysis of the paraformaldehyde fixed cells was carried out by FACScan flow cytometer with an excitation wavelength of 488 nm. R123 and propidium iodate fluorescence were determined for 15 000 cells, considering only viable cells (PI-excluding cells). Fluorescence data were shown on a three-decade log scale.

### Data analysis

All cytotoxicity data were modelled by non-linear regression using Kaleidagraph (Ablebeck Software, USA). Dose–response curves for cytotoxicity of drugs in absence or presence of the P-gp inhibitor GF120918 were analysed by non-linear curve fitting, using measured absorption of formazan produced by viable cells at 550 nm wavelength. Data were modelled with the general dose–response equation (DeLean et al, 1981):

$$Y = \left((a - d) / \left(1 + [X / C]^b\right)\right) + d$$

where Y is the absorption of formazan salts produced by viable cells at a molar concentration of cytotoxic drug X. The maximum of the curve is a, the minimum is d, the slope factor is b and C the drug concentration which inhibits the cell proliferation by 50%. For most curves a, b, C and d were the modelled parameters.

The effect of GF120918 to sensitize MES-Dx5 cells to cytotoxic drugs was expressed as % maximal shift of resistance, using the following formula:

$$= \left[ \frac{EC_{50}R \div EC_{50}(R+I)}{EC_{50}R \div EC_{50}(R+I_{max\ conc})} \right] \times 100$$

with the $EC_{50}$, the concentration of the cytotoxic drug required to inhibit cell proliferation by 50% for R the resistant cell line MES-Dx5, S the sensitive cell line MES-SA and I the P-gp inhibitor GF120918 at various concentrations. Ligand binding experiments were analysed by non-linear curve fitting as previously described (Malkhandi et al, 1995).

Means from independent experiments (*n*) are given with standard errors of mean, s.e.m. Statistical significance was analysed with the *t*-test.

### RESULTS

### Reversal of MES-Dx5 resistance to cytotoxics by GF120918

The wild-type MES-SA cells were subjected to a 3-day exposure to various cytotoxic agents and cell viability was assessed with the formazan-based MTT assay. The MES-SA cells were sensitive to vinblastine, taxotere and taxol with an $EC_{50}$ of 3 ± 1 nM ($n = 4$), 5 ± 1 nM ($n = 2$) and 5 ± 1 nM ($n = 5$) respectively, less sensitive to doxorubicin with an $EC_{50}$ of 58 ± 14 nM ($n = 5$) and etoposide with an $EC_{50}$ of 3003 ± 1006 nM ($n = 5$) (Table 1 and Figure 2 A–E).

The doxorubicin selected cell line MES-Dx5 exhibited the following resistance pattern compared to the parental cell line MES-SA: taxol 94-fold, taxotere 19-fold, vinblastine tenfold, doxorubicin ninefold and etoposide twofold resistance (Table 1 and Figure 2 A–E). One striking feature of these dose–response curves is the variation in the steepness of the slope, a topic usually ignored in the literature.

 © 1999 Cancer Research Campaign

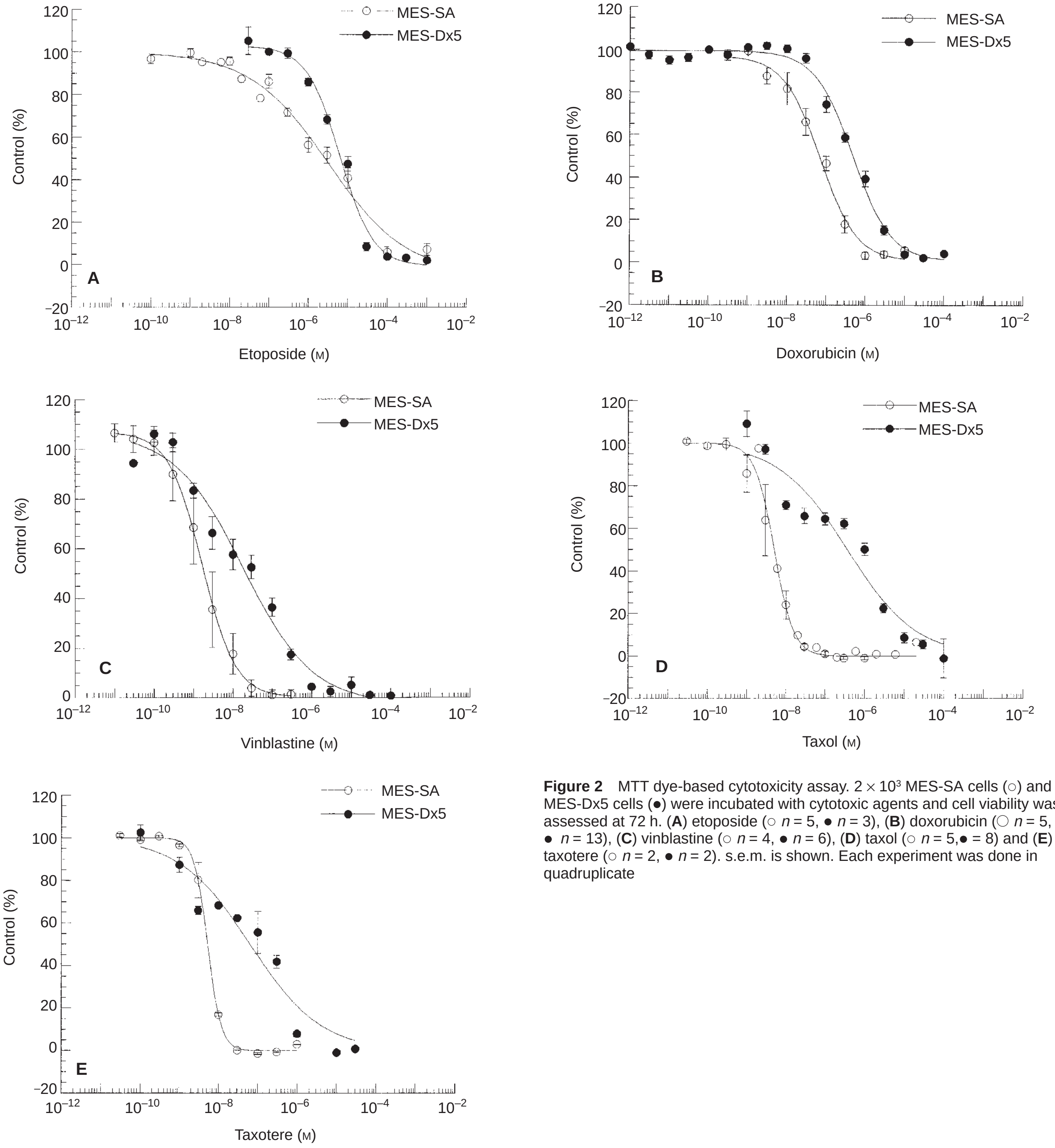

**Figure 2** MTT dye-based cytotoxicity assay. $2 \times 10^3$ MES-SA cells (○) and MES-Dx5 cells (●) were incubated with cytotoxic agents and cell viability was assessed at 72 h. (**A**) etoposide (○ $n$ = 5, ● $n$ = 3), (**B**) doxorubicin (○ $n$ = 5, ● $n$ = 13), (**C**) vinblastine (○ $n$ = 4, ● $n$ = 6), (**D**) taxol (○ $n$ = 5,● = 8) and (**E**) taxotere (○ $n$ = 2, ● $n$ = 2). s.e.m. is shown. Each experiment was done in quadruplicate

In MES-Dx5 cells a low slope was observed for the dose–response curve of all tubulin inhibitors (vinblastine < taxol < taxotere) (Table 1) compared to (i) doxorubicin and etoposide or (ii) the dose–response curves in MES-SA cells. The dose–response curves for taxanes in particular had slope factors of < 1.0 ($P$ < 0.0001), implying that proportional increases in the doses of taxanes have less effect than for drugs with steep dose–response curves such as doxorubicin.

The addition of the P-gp inhibitor GF120918 (100 nM) for 72 h did not affect sensitivity of the parental MES-SA cells to any of the five cytotoxics tested (results not shown). GF120918 alone did not produce a cytotoxic effect during the 72-h exposure in the MTT assay. An example of the sensitizing effect of GF120918 for taxol in MES-Dx5 cells is shown in Figure 3A. GF120918 concentrations of greater than 10 nM steepened the dose–response curves for taxol, vinblastine and taxotere. GF120918 was able to fully reverse resistance of the MES-Dx5 cells to the level of drug sensitivity shown by the parental MES-SA cells for all tested cytotoxic drugs. Based upon the analysis undertaken, the $EC_{50}$ of GF120918 to sensitize MES-Dx5 cells to taxol was 91 ± 23 nM ($n$ = 8) but 7 ± 2 nM ($n$ = 3) for etoposide (Table 2, Figure 3 B, C). The relative resistance to each cytotoxic agent correlated with the

© 1999 Cancer Research Campaign

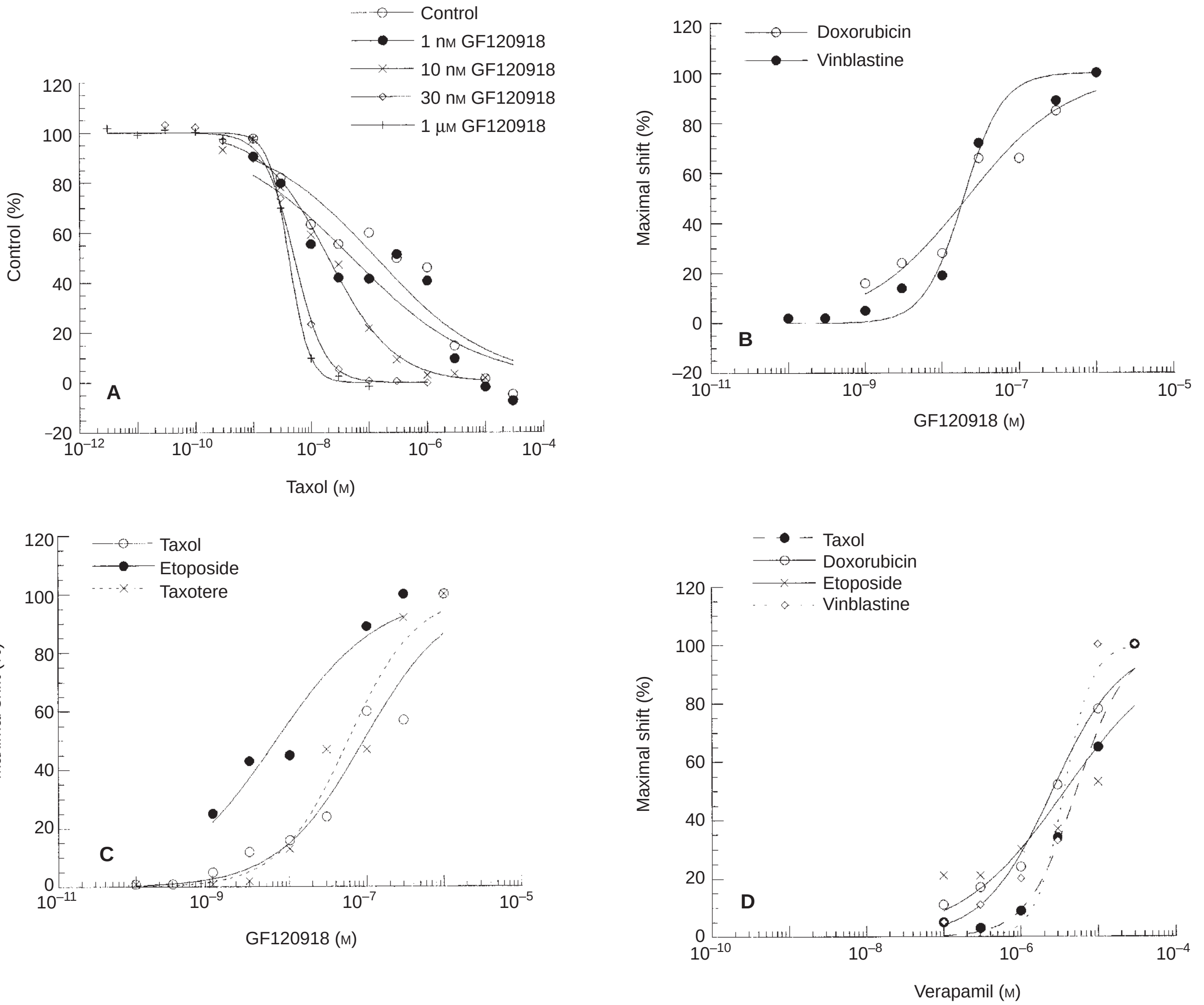

**Figure 3** Sensitization of MES-Dx5 cells by GF120918 or verapamil assessed with MTT dye-based cytotoxicity assay. Cell viability was assessed at 72 h. (**A**) MES-Dx5 cells were incubated with taxol (○), taxol and 1 nM GF120918 (●), 10 nM GF120918 (×), 30 nM GF120918 (◇) and 1 µM GF120918 (+). Data were from a single representative experiment done in quadruplicate. (**B** and **C**) Proportion of maximal shift of $EC_{50}$ produced by GF120918 for the cytotoxics: (**B**) (○) doxorubicin, (●) vinblastine, (**C**) (○) taxol, (●) etoposide and (×) taxotere ($n$ = 2–9, each experiment in quadruplicate). (**D**) Proportion of maximal shift of $EC_{50}$ produced by verapamil for the cytotoxics: (●) taxol, (○) doxorubicin, (×) etoposide and (◇) vinblastine ($n$ = 1–2, each experiment in quadruplicate)

**Table 2** Concentrations of GF120918 required to shift sensitivity of MES-Dx5 cells to cytotoxic drugs by 50% and to reverse accumulation deficits by 50%

| Drug | [GF120918], nM, shifts sensitivity by 50%[a] | [GF120918], nM, reverses accumulation-deficit by 50%[b] |
|---|---|---|
| Etoposide | 7 ± 2[c,d] | N/A |
| Doxorubicin | 21 ± 6 | 64 ± 21 |
| Vinblastine | 19 ± 3[c] | 48 ± 6 |
| Taxotere | 57 ± 14 | N/A |
| Taxol | 91 ± 24 | 37 ± 3 |

[a]Calculation of $EC_{50}$ of GF120918 which caused a 50% increase in sensitivity as assessed by cytotoxicity assay (MTT) is described in Materials and Methods. See Figure. 3A for an example of a representative experiment. [b]Radiolabelled cytotoxic drug accumulation assay were performed as described in Materials and Methods. Actual dose–response curves are shown in Figure 5. [c]$P < 0.05$ taxol vs other drug. [d]$P < 0.05$ taxotere vs other drug.

GF120918 concentration needed to restore 50% of sensitivity ($r$ = 0.9, $P$ = 0.03). To test if this observation is due to P-gp in MES-Dx5 cells or the effect of GF120918, we have performed reversal experiments with another P-gp inhibitor, verapamil, in MES-Dx5 cells which modulated resistance to taxol, doxorubicin, vinblastine and etoposide equipotently with an $EC_{50}$ of ~3 µM (Figure 3D).

### [$^3$H]-vinblastine, [$^3$H]-taxol and [$^{14}$C]-doxorubicin accumulation assays

The non-P-gp expressing MES-SA cells accumulated [$^3$H]-vinblastine, [$^3$H]-taxol and [$^{14}$C]-doxorubicin in a time-dependent manner. MES-Dx5 cells accumulated radiolabelled drugs with a similar time course, but accumulated 20% less [$^3$H]-taxol, 20% less [$^{14}$C]-doxorubicin and 35% less [$^3$H]-vinblastine at steady state. GF120918 did not increase the cellular content of

 *© 1999 Cancer Research Campaign*

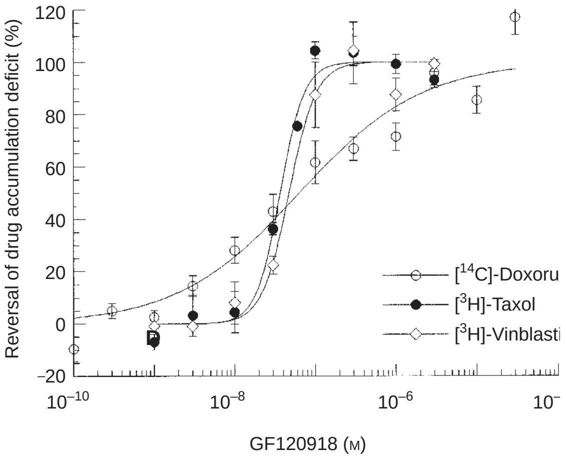

**Figure 4** Dose–response curves of GF120918 in the cellular accumulation assay. A total of 8 × $10^5$ MES-Dx5 cells were plated in each well and after 24 h of incubation 8 nM [$^{14}$C]-doxorubicin (○ *n* = 3), 0.1 nM [$^3$H]-vinblastine (◇ *n* = 3) or 0.7 nM [$^3$H]-taxol (● *n* = 2) and GF120918 were added and further incubated over 4 h. s.e.m. is shown. Each experiment was done in quadruplicate

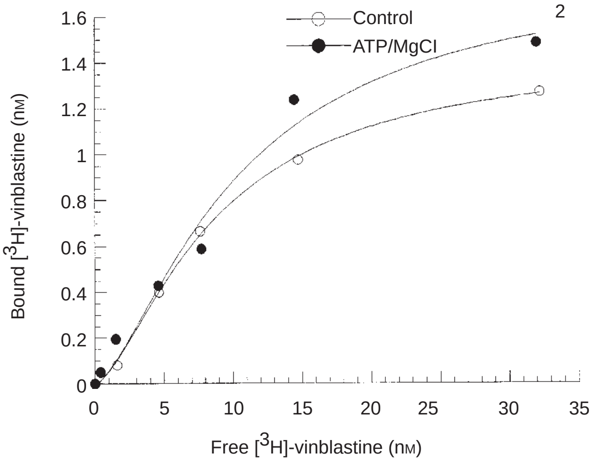

**Figure 6** Saturation isotherm of [$^3$H]-vinblastine binding to MES-Dx5 membrane preparations. Membrane preparation of MES-Dx5 (42 μg protein per assay) was incubated with 0.5–33 nM [$^3$H]-vinblastine and 3 μM vinblastine over 3 h (○). A total of 1 mM ATP and 3 mM $MgCl_2$ were added as indicated (●). Vinblastine binding to P-gp in MES-Dx5 cells achieved a $B_{max}$ = 8.6 ± 0.4 pmol mg$^{-1}$ protein and a $K_D$ = 8.7 ± 0.7 nM. In the presence of ATP and $MgCl_2$ the modelled parameters were $B_{max}$ = 10.9 ± 2.1 pmol mg$^{-1}$ protein and a $K_D$ = 10.3 ± 3.4 nM. Data shown are from a single representative experiment done in duplicate

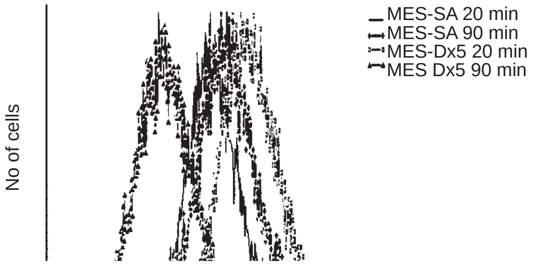

**Figure 5** FACS analysis of rhodamine accumulation and retention in MES-Dx5 and MES-SA cells. A total of 1 × $10^6$ MES-SA or MES-Dx5 cells were exposed to the fluorescent dye rhodamine followed by a 1.5-h long period of drug efflux. Cells were stained with propidium iodate, fixed with paraformaldehyde and sorted by FACS analysis. Then, 15 000 viable cells were collected using a 3-decade log scale. The P-gp expressing MES-Dx5 cells resulted in a lower rhodamine accumulation following the efflux phase. A narrow, single peak indicated a single population of P-gp expressing cells. The results of a representative experiment are shown radiolabelled cytotoxics in the sensitive MES-SA cells (data not shown). However in MES-Dx5 cells, at a saturating concentration of 1 μM, GF120918 increased the cellular accumulation of all three drugs by 30% at 4 h. In MCF-7/ADR cells, a breast cancer cell line selected in doxorubicin with sixfold higher expression of P-gp, 1 μM GF120918 increased the uptake of [$^{14}$C]-doxorubicin by 240% (data not shown).

A dose-dependent effect of GF120918 existed for all three radiolabelled cytotoxic drugs (Figure 4). The $EC_{50}$ for the reversal of the accumulation deficit by GF120918 in MES-Dx5 cells ranged from 37–64 nM (Table 2).

### Gel electrophoresis and Western blotting of membrane preparations

The monoclonal antibody NCL-JSB-1 could detect P-gp in Western blots of membranes prepared from MES-Dx5 cells (limit of detectability 10 μg membrane protein) but did not in MES-SA membranes. The calculated molecular weight for P-gp in MES-Dx5 cells was 156 kDa (data not shown).

### FACS analysis of P-gp expression

FACS analysis of rhodamine accumulation and its efflux was used to determine if the MES-Dx5 cells consisted of more than one population. MES-Dx5 cells were found to consist of a single-cell population with regard to rhodamine efflux (Figure 5).

### Membrane binding assay

Saturation analysis of [$^3$H]-vinblastine binding in MES-Dx5 cell membrane preparations revealed a $B_{max}$ of 5.2 ± 1.6 pmol mg$^{-1}$ protein and a $K_D$ of 6.8 ± 1.4 nM. The addition of ATP and $MgCl_2$ had only a minor effect on [$^3$H]-vinblastine binding (Figure 6).

[$^3$H]-vinblastine binding to membrane preparations of MES-SA was not displaceable by cyclosporin A (Figure 7A), a well described, competitive inhibitor of [$^3$H]-vinblastine binding to P-gp (Ferry et al, 1992). Cyclosporin A displaceable [$^3$H]-vinblastine binding to P-gp in MES-Dx5 membrane preparations was proportional to the amount of protein used (up to 300 μg). Cyclosporin A inhibited [$^3$H]-vinblastine binding to P-gp to the level of non-specific binding in MES-SA cells.

Inhibition of [$^3$H]-vinblastine binding to MES-Dx5 membranes was assessed using several cytotoxic agents and P-gp inhibitors. Doxorubicin was a weak inhibitor with a $K_i$ = 6940 ± 2100 nM followed in increasing potency by verapamil ($K_i$ = 660 ± 350 nM), taxol ($K_i$ = 400 ± 135 nM) and GF120918 ($K_i$ = 5 ± 1 nM) (Figure 7B). Taxol, an allosteric inhibitor of [$^3$H]-vinblastine binding (Ferry et al, 1994), could displace only 40% of [$^3$H]-vinblastine bound to P-gp.

*© 1999 Cancer Research Campaign* 

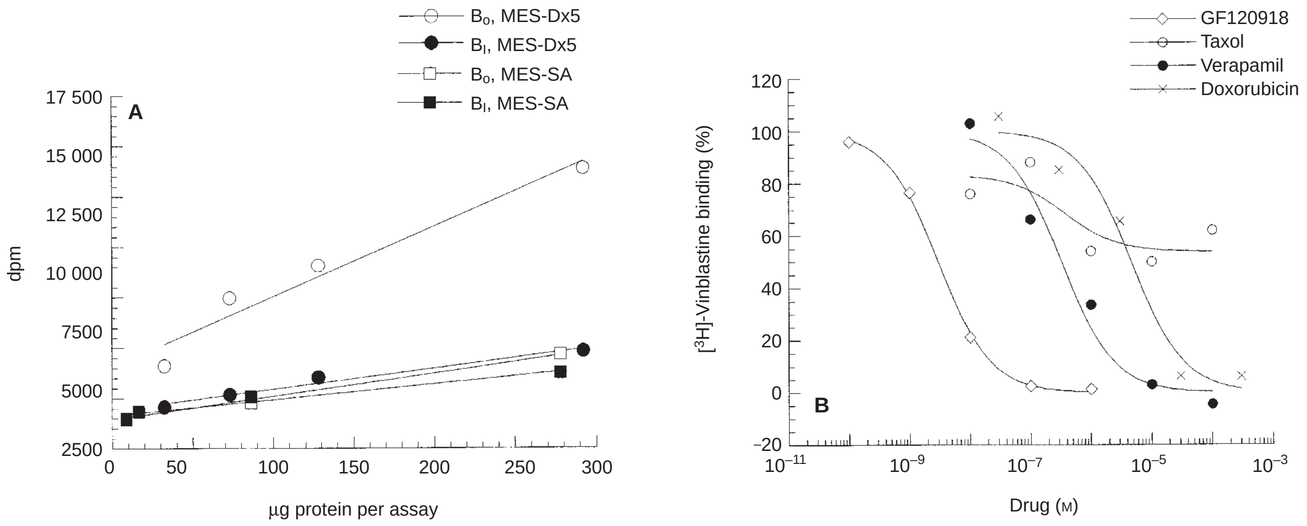

**Figure 7** [$^3$H]-vinblastine binding studies using cell surface membrane preparations of MES-SA and MES-Dx5 cells. (**A**) Cell surface membrane preparations (9–300 µg protein) were incubated with 7.6 nM [$^3$H]-vinblastine ($B_o$) and 10 µM cyclosporin A ($B_1$) for 2 h. MES-SA cells exhibited only non-specific binding of [$^3$H]-vinblastine. Binding of [$^3$H]-vinblastine to MES-Dx5 membranes increased in a protein dependent manner up to 300 µg protein. Cyclosporin A inhibited [$^3$H]-vinblastine binding to P-gp to the level of non-specific binding in MES-SA cells. (**B**) Inhibition of [$^3$H]-vinblastine binding to P-gp in MES-Dx5 membrane preparations by cytotoxic agents and P-gp inhibitors. Membrane preparations were incubated with 5 nM [$^3$H]-vinblastine and doxorubicin (×), taxol (○), verapamil (●) or GF120918 (◇) over 3 h. The $K_i$ values were as follows: doxorubicin = 112 µM, taxol = 96 nM, verapamil = 1.7 µM and GF120918 = 5 nM. Data shown are from a single representative experiment done in duplicate

**Table 3** Effect of protein binding on the potency of P-gp inhibitors using the $EC_{50}$ (nM) of taxol in P-gp-expressing MCF-7/ADR cells and the wild-type MCF-7 WT cells (MTT dye based cytotoxicity assay after 72-h exposure to drugs)

| Drug | 10% FCS | | 50% FCS | | Ratio of $EC_{50}$s[a] |
|---|---|---|---|---|---|
| | $EC_{50}$, nM | *n* | $EC_{50}$, nM | *n* | |
| None (MCF-7/WT) | 6 ± 2 | 3 | 5 ± 3 | 3 | 0.84 |
| None[b] | 8500 ± 1400 | 8 | 4600 ± 800 | 12 | 0.54[c] |
| GF120918 (0.1 µM)[b] | 8 ± 2 | 3 | 22 ± 5 | 3 | 2.6 ± 1.5 |
| PSC 833 (2 µM)[b] | 52 ± 22 | 3 | 980 ± 210 | 3 | 19 ± 6[d] |
| CGP 41251 (2 µM)[b] | 102 ± 56 | 3 | 3055 ± 400 | 3 | 30 ± 12[d] |
| Dexniguldipine (1 µM)[b] | 35 ± 8 | 3 | 1290 ± 37 | 3 | 37 ± 9[d] |
| CP100356 (1 µM)[b] | 81 ± 20 | 3 | 7120 ± 1800 | 3 | 88 ± 22[d] |

[a]Calculated with the formula Equation 3. [b]P-gp expressing MCF-7/ADR cells. [c]$P < 0.05$ $EC_{50}$ 50% FCS vs $EC_{50}$ 10% FCS; [d]$P < 0.05$ ratio of $EC_{50}$s for GF120918 vs other inhibitors.

### Effect of a high concentration of fetal calf serum on the potency of GF120918 in MCF-7/ADR cells

MES-Dx5 cells grew poorly in medium supplemented with 50% FCS. Therefore MCF-7/ADR cells and their drug-sensitive wild-type MCF-7 cells were used to explore the effect of a high protein concentration on the potency of GF120918 (MTT assay). The cells were incubated with the cytotoxic drug taxol in the presence of a series of potent P-gp inhibitors and either 10% or 50% FCS. The data are shown in Table 3. Although all P-gp inhibitors were less potent in 50% FCS, this effect was significantly smaller ($P < 0.05$, *t*-test) for GF120918 (2.6 ± 1.5-fold) compared to CP100356 (88 ± 22-fold), dexniguldipine (37 ± 9-fold), CGP 412251 (30 ± 12-fold) or PSC833 (19 ± 6-fold).

### DISCUSSION

MES-Dx5 cells were derived from a human uterine sarcoma cell line, MES-SA after long-term selection in 500 nM doxorubicin (Harker et al, 1983; Harker and Sikic, 1985). The resistance factors to doxorubicin, vinblastine and etoposide in the 72-h MTT cytotoxicity assay we report were lower than those previously reported for the 1-h drug exposure in a colony-forming assay (Harker and Sikic, 1985). This is not surprising for tubulin-binding drugs and etoposide which show marked cell-cycle/phase dependency. After a 72-h exposure of MES-Dx5 cells to cytotoxic drugs, a resistance pattern similar to the one we have observed has been described (Gosland et al, 1989).

MES-Dx5 cells expressed P-gp as assessed by Western blotting and [$^3$H]-vinblastine binding, which allowed quantification of the number of P-gp molecules per mg protein. In the drug sensitive clone MES-SA P-gp could not be detected by either method. The density of [$^3$H]-vinblastine binding sites in MES-Dx5 membrane preparations was about 25% that of MCF-7/ADR cells (Ferry et al, 1995). This correlates with the higher resistance of MCF-7/ADR cells to e.g. doxorubicin (200-fold) and taxol (1000-fold) (MA Russell and DR Ferry, unpublished data).

 © 1999 Cancer Research Campaign

One striking feature of the dose–response curves of the cytotoxic drugs in MES-Dx5 cells was the variation in their slopes. Thus the slopes of the dose–response curves for taxanes and vinblastine were significantly lower than for doxorubicin. When the P-gp inhibitor GF120918 (Hyafil et al, 1993), an acridone-carboxamide, was added to these cells, the slopes of the dose–response curves for taxanes and vinblastine became steeper, reaching the same level as those observed in the parental MES-SA cells.

One possible explanation for this phenomenon could be, that MES-Dx5 cells consisted of two or more populations of cells expressing different amounts of P-gp. However, in a rhodamine FACS analysis (using the method described by Davies et al (1996)), a single population of MES-Dx5 cells was detected. Other explanations needed to be further considered, including heterogeneity of tubulin expression due to mutations in microtubules. Mutations in tubulin subunits were described in a series of mutant Chinese hamster ovary cell lines which rendered the cells resistant to taxol (Schibler and Cabral, 1986). Assuming the level of resistance to taxol in MES-Dx5 cells was due to the combined effects of P-gp and mutated microtubulin, the inhibition of P-gp should only lead to a shift of the dose–response curve to the left, e.g. concentrations of cytotoxic drugs achieve higher level of cytotoxicity but not to an alteration in the steepness of the dose–response curve. This was clearly not the case since the P-gp inhibitor GF120918 fully sensitized MES-Dx5 and additionally increased the slope of the dose–response curve for all tubulin binding drugs, taxol, taxotere and vinblastine.

MES-Dx5 cells were fully sensitized to a variety of natural product cytotoxics with nM concentrations of the recently described potent P-gp inhibitor GF120918, achieving the same sensitivity as the parental MES-SA cells. GF120918 acted potently on P-gp with concentrations as low as 1 nM, exhibiting a measurable reversal of resistance in the MTT assay. However, fivefold higher concentrations of GF120918 were required to reverse resistance to taxol by 50% compared to doxorubicin and vinblastine. Similarly an eightfold higher concentration of GF120918 was required to reverse resistance to taxotere by 50% compared to etoposide. If GF120918 bound to a common site, 'plugging' P-gp, the $EC_{50}$ to reverse resistance expressed in the cytotoxicity assay and the $EC_{50}$ to increase the cellular accumulation of drugs, should be equal. A number of possible explanations for this phenomenon are discussed below.

### *The potency of GF120918 to reverse resistance relates to the affinity of cytotoxics to bind to P-gp*

We know that [$^3$H]-GF120918 can bind saturably and with a 0.8 nM $K_D$ to human P-gp (Ferry et al, 1996). Theoretically the explanation may lie in the relative affinities of the cytotoxic drugs for P-gp. Thus, if drug A (e.g. taxol or vinblastine) has a very high affinity for P-gp relative to drug B (e.g. doxorubicin or etoposide) and both are present at equal concentrations, more GF120918 will be needed to inhibit the binding of drug A by 50%. However, there was no clear relationship between the potency to modulate a given cytotoxic drug and the affinity to bind to P-gp. Furthermore, since the $EC_{50}$ for GF120918 to reverse drug resistance was higher for taxol than for vinblastine this explanation must be flawed. These observations therefore imply that P-gp cannot be regarded as a simple receptor at which drugs compete.

### *The potency of GF120918 to reverse resistance relates to binding affinities for different P-gp drug acceptor sites.*

Accepting that taxol and GF120918 both bind to P-gp, one possibility is that GF120918 has a lower affinity for a taxane-selective binding domain of P-gp. This would be revealed by higher concentrations of GF120918 required to reverse the [$^3$H]-taxol accumulation deficit. However the $EC_{50}$ of GF120918 to reverse the [$^3$H]-taxol accumulation deficit was 37 ± 3 nM and not significantly higher than for the other drugs.

One reason for the failure of the above model to explain why GF120918 is less potent in modulating resistance to taxol and taxotere compared to the other cytotoxic drugs may be that the assumptions underlying the models are incorrect. The mechanisms of permeation of P-gp by cytotoxics is poorly characterized. The initial rate of transport and turnover numbers of P-gp are not known for any substrate.

The model assumes that GF120918, taxol and the other drugs bind to a common drug acceptor site of P-gp. Whilst previous data was interpreted as suggesting that P-gp has a unitary drug acceptor site (Raviv et al, 1990), more recent data suggest that P-gp has more than one drug acceptor site which may even be allosterically coupled (Spoelstra et al, 1992; Ferry et al, 1994; Boer et al, 1996; Day et al, 1997).

Thus if GF120918 has a lower affinity for the taxol drug acceptor site versus that for vinblastine, the potency to modulate resistance would be lower. This remains to be tested using [$^3$H]-GF120918 as a radioligand in binding studies.

### *The effect of other membrane drug transporters*

Resistance to etoposide and doxorubicin can be conferred by the expression of the multidrug-resistance associated protein, MRP1 (Cole et al, 1992), a membrane transporter protein. Both, the wild-type MES-SA cells and the P-gp-positive clone MES-Dx5 express similar levels of MRP1 (Chen et al, 1994). Additionally, it was demonstrated that the lung cancer cell line COR-L23 R (Barrand et al, 1994), which expresses MRP1 but not P-gp, was not sensitized to doxorubicin by 1 μM GF120918 (HCL Traunecker and DR Ferry, unpublished data). Therefore, the effect of GF120918 is unlikely to be due to the inhibition of MRP1.

The major vault protein, LRP, a transporter protein present in vesicular membranes has been implicated in resistance to doxorubicin (Scheper et al, 1993). Although LRP has been detected in parental MES-SA cells, no expression was detectable in the MES-Dx5 cells (Chen et al, 1994).

### *The resistance factor correlates with the concentration of GF120918 required to modulate the resistance*

The striking correlation between the $EC_{50}$ of GF120918 to reverse resistance in the cytotoxicity assay and the resistance factor of each cytotoxic agent ($r$ = 0.9) suggests that the potency of GF120918 relates closely with the resistance factor of the cytotoxic drug. This implies, that a given concentration of GF120918 induces a given fractional modulation of resistance. It is difficult to envisage how this could occur at the molecular level.

Although there is no direct evidence, it is mechanistically plausible that GF120918 is a more potent modulator of resistance to vinblastine, doxorubicin and etoposide than the taxanes probably as a consequence of the pattern of binding to P-gp and subsequent inhibition of substrate transport by P-gp in MES-Dx5 cells. Experiments aimed at measuring direct binding of [$^3$H]-taxol and

© 1999 Cancer Research Campaign

[$^3$H]-GF120918 to P-gp may cast some light and give some clues as to the molecular mechanisms underlying the phenomenon observed in the cytotoxicity assays.

GF120918 is the most potent P-gp inhibitor described (Hyafil et al, 1993) and retains most of its activity at high protein concentrations (50% FCS). All other tested P-gp inhibitors, i.e. CP100356, dexniguldipine, CGP 412251 and PSC833 were significantly less potent in the presence of 50% FCS. This finding is important for clinical studies in view of the high protein concentrations present in whole blood. In this manuscript detailed pharmacological data regarding the reversal of resistance to vinblastine, doxorubicin, etoposide, taxotere and taxol by GF120918 have been documented for the first time. The results have significant implications on a number of levels. Functional studies of P-gp, particularly binding and transporting of cytotoxic agents and P-gp inhibitors are not yet sophisticated enough to estimate initial rates of transport or turnover rates for any substrate. The relatively low expression of P-gp in cells was a major problem which some groups recently overcame by purifying P-gp (Shapiro et al, 1995; Callaghan et al, 1997). This may allow to re-address these fundamental questions in the future.

GF120918 has entered phase I clinical trials in combination with doxorubicin (Ferry et al, 1998; Planting et al, 1998). The serum levels achieved are known to reverse the P-gp mediated efflux of rhodamine in CD56 cells (natural killer cells) in normal volunteers (Kerr et al, 1996) and the full trial data will be presented soon (Ferry et al, in preparation).

In terms of the future clinical application, it may prove important that the resistance to doxorubicin could be modulated at lower concentrations of GF120918 compared to the taxanes, taxol and taxotere but remains to be tested.

### ACKNOWLEDGEMENTS

This work has been supported by a Price-Hall Fellowship from the Special Trustees of the Former United Birmingham Hospitals and by grants from The Birmingham Children's Hospital Centenary Research Fund and the Department of Paediatric Oncology at the Birmingham Children's Hospital NHS Trust. GlaxoWellcome provided GF120918. We also wish to thank Michael Russell for contributing the data on the MCF-7/ADR and wild-type cell lines.

### REFERENCES

Barrand MA, Heppel-Parton AC, Wright KA, Rabbits PH and Twentyman PR (1994) A 190k protein overexpressed in non-P-glycoprotein containing MDR cells and its relation to the MRP gene. *J Natl Cancer Inst* **86**: 110–117

Boer R, Dichtl M, Borchers C, Ulrich WR, Marecek JF, Prestwich GD, Glossmann H and Striessing J (1996) Reversible labeling of a chemosensitizer binding domain of P-glycoprotein with a novel 1,4-dihydropyridine drug transport inhibitor. *Biochemistry* **35**: 1387–1396

Callaghan R, Berridge G, Ferry DR and Higgins CF (1997) The functional purification of P-glycoprotein is dependent on maintenance of a lipid protein inter-face. *Biochem Biophys Acta Biomembranes* **1328**: 109–124

Chen G, Jaffrezou J-P, Fleming WH, Duran GE and Sikic BI (1994) Prevalence of multidrug resistance related to activation of the mdr1 gene in human sarcoma mutants derived by single-step doxorubicin selection. *Cancer Res* **54**: 4980–4987

Cole SPC, Bhardwaj G, Gerlach JH, Mackie JE, Grant CE, Almquist KC, Stewart AJ, Kurz E, Duncan AMV and Deeley RG (1992) Over-expression of a transporter gene in a multidrug resistant human lung cancer cell line. *Science* **258**: 1650–1653

Davies R, Budworth J, Riley J, Snowden R, Gescher A and Gant T (1996) Regulation of P-glycoprotein 1 and 2 gene expression and protein activity in two MCF-7/Dox cell line subclones. *Br J Cancer* **73**: 307–315

Day S, Ramachandra M, Pastan I, Gottesman MM and Ambudkar SV (1997) Evidence for two non-identical drug-interaction sites in the human P-glycoprotein. *Proc Natl Acad Sci USA* **94**: 10594–10599

DeLean A, Hancock AA and Lefkowitz RJ (1981) Validation and statistical analysis of a computer modelling method for quantitative analysis of radioligand binding data for mixtures of pharmacological receptor subtypes. *Mol Pharmacol* **21**: 5–16

Endicott JA and Ling V (1989) The biochemistry of P-glycoprotein-mediated multidrug resistance. *Annu Rev Biochem* **58**: 137–171

Ferry DR, Russell MA and Cullen MH (1992) P-glycoprotein possesses a 1,4-dihydropyridine-selective drug acceptor site which is allosterically coupled to a vinca alkaloid-selective binding site. *Biochem Biophys Res Commun* **188**: 440–445

Ferry DR, Russell MA and Kerr DJ (1994) [$^3$H]-Taxol binds to a drug acceptor site which is allosterically coupled to the vinblastine-selective site of P-glycoprotein. *Proc Am Assoc Cancer Res* **35**: 348

Ferry DR, Malkhandi J, Russell MA and Kerr DJ (1995*a*) Dexniguldipine-HCl is a potent allosteric modulator of [$^3$H]-vinblastine binding to P-glycoprotein of MCF-7 ADR breast cancer cell membranes. *Biochem Pharmacol* **49**: 1851–1861

Ferry DR, Malkhandi PJ, Russell MA and Kerr DJ (1995*b*) Allosteric regulation of [$^3$H]vinblastine binding to P-glycoprotein of MCF-7 ADR cells by dexniguldipine. *Biochem Pharmacol* **49**: 1851–1861

Ferry DR, Russell MA, Kerr DJ, Correa ID and Prakash SR (1996) [$^3$H]-GG918 (GF120918) binds with positive co-operativity to human P-glycoprotein with a nM dissociation constant. *Proc Am Assoc Cancer Res* **37**: abstract 2246

Ferry D, Moore M, Bartlett NL, Fyfe D, Oza G, Fracasso PM, Kersey K, Wissel PS, Jewell RC and Paul EM (1998) Phase I and pharmacokinetic (PK) study targeting a 500 ng/ml plasma concentration of the potent multidrug resistance (MDR) modulator GF120918 (GF) with doxorubicin (DOX) in patients with advanced solid tumors. *Proc Am Soc Clin Oncol* **17**: 240a

Ford JM and Hait WN (1990) Pharmacology of drugs that alter multidrug resistance in cancer. *Pharmacol Rev* **42**(3): 155–199

Gosland MP, Lum BL and Sikic BI (1989) Reversal by cefoperazone of resistance to etoposide, doxorubicin, and vinblastine in multidrug resistant human sarcoma line. *Cancer Res* **49**: 6901–6905

Gottesman MM and Pastan P (1993) Biochemistry of multidrug resistance mediated by the multidrug transporter. *Annu Rev Biochem* **62**: 385–427

Harker WG and Sikic BI (1985) Multidrug (pleiotropic) resistance in doxorubicin selected variants of the human sarcoma cell line MES-SA. *Cancer Res* **45**: 4091–4096

Harker WG, MacKintosh FR and Sikic BI (1983) Development and characterization of a human sarcoma cell line, MES-SA, sensitive to multiple drugs. *Cancer Res* **43**: 4943–4950

Hyafil F, Vergely C, Du VP and Grand PT (1993) In vitro and in vivo reversal of multidrug resistance by GF120918, an acridonecarboxamide derivative. *Cancer Res* **53**(19): 4595–4602

Kajiji S, Dreslin JA, Grizzuti K and Gros P (1994) Structurally distinct MDR modulators show specific pattern of reversal against P-glycoproteins bearing unique mutations at serine (939/941). *Biochem* **33**(17): 5041–5048

Malkhandi PJ, Ferry DR, Boer R and Kerr DJ (1995) P-glycoprotein has a drug acceptor site for 1,4-dihydropyridines which is localised on an intracellular domain. *Proc Am Assoc Cancer Res* **36**: 332

Miller TP, Grogan TM, Dalton WS, Spier CM, Scheper RJ and Salmon SE (1991) P-glycoprotein expression in malignant lymphoma and reversal of clinical drug resistance with chemotherapy plus high dose verapamil. *J Clin Oncol* **9**: 17–24

Mosmann T (1983) Rapid colorimetric assay for cellular growth and survival: application to proliferation and cytotoxicity assays. *J Immunol Methods* **65**: 55–63

Planting A, van der Gaast A, Sparreboom A, van der Burg MEL, de Boer M, Wissel PS, Jewell RC, Paul EM and Verweij J (1998) Phase I and pharmacokinetic (PK) study targeting a 100 ng/ml plasma concentration of the potent multidrug resistance (MDR) modulator GF120918 (GF) with doxorubicin (DOX) in patients with advanced solid tumors. *Proc Am Soc Clin Oncol* **17**: 199a

Raviv Y, Poland HB, Bruggermann EP, Pastan I and Gottesman MM (1990) Photosensitive labeling of a functional multidrug transporter in living drug resistant tumour cells. *J Biol Chem* **265**: 3975–3980

Sambrook J, Fritsch EF and Maniatis T (1989). Transfer of proteins from SDS-polyacrylamide gels to solid supports: immunological detection of

 © 1999 Cancer Research Campaign immobilized proteins (Western blotting). In: *Laboratory Cloning, a Laboratory Manual*, Nolan C (ed), pp. 18.60–18.75. Cold Spring Harbor Laboratory Press: New York

Scheper RJ, Broxterman HJ, Scheffer GL, Kaaijk P, Dalton WS, van Heijningen THM, van Kalken CK, Slovak ML, de Vries EGE, van der Valk P, Meijer CJLM and Pinedo HM (1993) Overexpression of a $M_r$ 110 000 vesicular protein in non-P-glycoprotein-mediated multidrug resistance. *Cancer Res* **53**: 1475–1479

Schibler MJ and Cabral F (1986) Taxol-dependent mutants of chinese hamster ovary cells with alterations in alpha-tubulin and beta-tubulin. *J Cell Biol* **102**: 1522–1531

Shapiro AB, Ling V and Doige CA (1995) Reconstitution of drug transport by purified P-glycoprotein. *J Biol Chem* **270**: 16167–16175

Sonneveld P, Durie BGM, Lokhorst HM, Marie J-P, Solbu G, Zittoun R, Lowenberg B and Nooter K (1992) Modulation of multidrug resistant myeloma by cyclosporin. *Lancet* **340**: 255–258

Spoelstra EC, Westerhoff HV, Dekker H and Lankelma J (1992) Kinetics of daunorubicin transport by P-glycoprotein of intact cells. *Eur J Biochem* **207**: 567–579

Witherspoon SM, Emerson DL, Kerr BM, Lloyd TL, Dalton WS and Wissel PS (1996) Flow cytometric assay of modulation of P-glycoprotein function in whole blood by the multidrug resistance inhibitor GG918. *Clin Cancer Res* **2**: 7–12

*© 1999 Cancer Research Campaign*
